# Long-term prosthetic-associated subclinical thrombotic events evaluation by cardiac CTA after transcatheter aortic valve implantation: incidence and outcomes

**DOI:** 10.1186/s13244-024-01681-0

**Published:** 2024-05-30

**Authors:** Qijing Zhou, Jiaqi Wen, Qifeng Zhu, Jiaqi Fan, Xiaojun Guan, Xinyi Chen, Yuxin He, Yuchao Guo, Jubo Jiang, Xinfa Ding, Zhaoxia Pu, Zhaoxu Huang, Cheng Li, Minming Zhang, Xianbao Liu, Xiaojun Xu, Jian’an Wang

**Affiliations:** 1 Department of Radiology, Second Affiliated Hospital Zhejiang University School of Medicine, Hangzhou, People’s Republic of China; 2 Department of Cardiology, Second Affiliated Hospital Zhejiang University School of Medicine, Hangzhou, People’s Republic of China; 3 Department of Epidemiology, Harvard T.H. Chan School of Public Health, Boston, MA USA; 4 Department of Echocardiography, Second Affiliated Hospital Zhejiang University School of Medicine, Hangzhou, People’s Republic of China; 5 Department of Nursing, Second Affiliated Hospital Zhejiang University School of Medicine, Hangzhou, People’s Republic of China; 6 Zhejiang University School of Medicine, Hangzhou, People’s Republic of China

**Keywords:** Subclinical thrombotic, Transcatheter aortic valve implantation, Computed tomography, Long-term, Outcome

## Abstract

**Objective:**

To observe prosthetic-associated subclinical thrombotic events (PASTE) after transcatheter aortic valve implantation (TAVI) by cardiac CTA, and assess their impact on long-term patient outcomes.

**Materials:**

We prospectively and consecutively enrolled 188 patients with severe aortic stenosis treated with TAVI from February 2014 to April 2017. At 5 years, 61 of 141 survived patients who had completed annual follow-up CTA (≥ 5 years) were included. We analyzed PASTE by CTA, including hypoattenuated leaflet thickening (HALT), sinus filling defect (SFD), and prosthesis filling defect (PFD). The primary outcome was a major adverse cardiovascular composite outcome (MACCO) of stroke, cardiac re-hospitalization, and bioprosthetic valve dysfunction (BVD); the secondary outcomes were bioprosthetic hemodynamics deterioration (PGmean) and cardiac dysfunction (LVEF).

**Results:**

During a median follow-up time of 5.25 years, long-term incidence of HALT, SFD, and PFD were 54.1%, 37.7%, and 73.8%, respectively. In the primary outcome, SFD and early SFD were associated with the MACCO (SFD: *p* = 0.005; early SFD: *p* = 0.018), and SFD was a predictor of MACCO (HR: 2.870; 95% CI: 1.010 to 8.154, *p* = 0.048). In the secondary outcomes, HALT was associated with increased PGmean (*p* = 0.031), while persistent HALT was correlated with ΔPGmean (β = 0.38, *p* = 0.035). SFD was negatively correlated with ΔLVEF (β = −0.39, *p* = 0.041), and early SFD was negatively correlated with LVEF and ΔLVEF (LVEF: *r* = −0.50, *p* = 0.041; ΔLVEF: *r* = −0.53, *p* = 0.030).

**Conclusions:**

PASTE were associated with adverse long-term outcomes, bioprosthetic hemodynamics deterioration, and cardiac dysfunction. In particular, SFD was a predictor of MACCO and may be a potential target for anticoagulation after TAVI (NCT02803294).

**Registration:**

URL: https://www.clinicaltrials.gov; Unique identifier: NCT02803294.

**Critical relevance statement:**

PASTE, especially SFD, after TAVI based on cardiac CTA findings impacts the long-term outcomes of patients which is a predictor of long-term major adverse outcomes in patients and may be a potential target for anticoagulation after TAVI.

**Key Points:**

- Transcatheter aortic valve implantation is being used more often; associated subclinical thromboses have not been thoroughly evaluated.
- Prosthetic-associated subclinical thrombotic events were associated with adverse outcomes, bioprosthetic hemodynamics deterioration, and cardiac dysfunction.
- Studies should be directed at these topics to determine if they should be intervened upon.

**Graphical Abstract:**

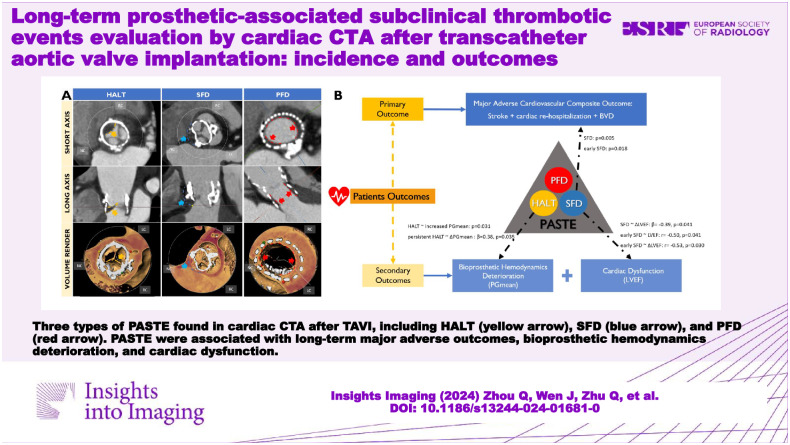

## Introduction

Transcatheter aortic valve implantation (TAVI) is increasingly used in patients with symptomatic severe aortic stenosis (AS), as it has been proven to be a safe and effective treatment option across the entire spectrum of surgical risk [1–3]. Long-term patient outcomes after TAVI have become increasingly important, especially with the PARTNER 3 and Evolut clinical cohort studies that achieved milestones progress in 2019, it shifts to younger, lower-risk patients with longer life expectancies [4–7].

Prosthesis-associated subclinical thrombotic events (PASTE) after TAVI are an important imaging finding in cardiac computed tomography angiography (CTA) follow-up after TAVI. Previously, studies mainly focused on subclinical lobular thrombosis (SLT), which was defined as hypoattenuated leaflet thickening (HALT) diagnosed in cardiac CTA [8–10]. It has been shown that it is associated with clinical valve thrombosis, TIA and stroke, structural valve degeneration, or symptomatic hemodynamic valve deterioration [11–15]. However, previous HALT evaluations based on cardiac CTA results were early assessments, with CTA performed 30 days to 1 year after TAVI, while long-term follow-up evaluations of HALT were insufficient.

Moreover, there are two other types of PASTE that can be found on cardiac CT after TAVI, aortic sinus thrombosis and prosthesis thrombus, which can displayed as a hypoattenuated filling defect in the aortic sinus (sinus filling defect, SFD) or inside the mental stent structure of prosthesis (prosthesis filling defect, PFD). These have not received sufficient attention. A few previous studies have noticed the reduced flow with stasis in the primary aortic sinus and the phenomenon of SFD after TAVI, but failed to explore possible effects on long-term clinical outcomes [16]. There have been plenty of previous studies on PFD after SAVR, as it is a cause of hemodynamic change and prosthetic valve dysfunction after SAVR [17–19]. However, the finding of PFD after TAVI and its clinical significance has not been reported. Therefore, a more systematic and comprehensive assessment of PASTE after TAVI and its clinical impact may be required.

In this study, we aimed to observe the incidence of PASTE in the long-term follow-up, including HALT, SFD, and PFD, and evaluate the impact of PASTE on long-term patient outcomes.

## Methods

### Study design and patient population

The TORCH (Transcatheter Aortic Valve Replacement Single Center Registry in Chinese Population) registry (NCT02803294) is a single-center prospective cohort study in the Chinese population. The study was approved by the medical ethics committee and carried out according to the principles of the Declaration of Helsinki. All patients provided written informed consent for TAVI and the use of anonymous clinical, procedural, and follow-up data for research.

For this study, we consecutively and prospectively collected 188 severe AS patients treated with TAVI from February 2014 to April 2017, who were determined by an interdisciplinary heart team. Since this study was focused on the evaluation of the long-term incidence of PASTE and its impact on patient long-term outcomes, patients who completed long-term annual follow-up and annual cardiac CTA after TAVI were ultimately enrolled in this study. Standard exclusion criteria for cardiac CTA were applied, including severe allergy to iodine-containing contrast material, impaired renal function (creatinine ≥ 1.5 mg/dl), arrhythmia, and severe respiratory or cardiac failure. In addition, all patients received a standard annual follow-up procedure, including cardiac CT and transthoracic echocardiography (TTE), which were performed as a routine annual follow-up.

### CT acquisition

Cardiac CTA was performed on 2nd or 3rd generation dual-source 128-slice or 192-slice CT scanners (SOMATOM Flash and Force, Siemens Healthineers, Forchheim, Germany). It was performed at 100/Sn140 (SOMATOM Flash) or 90/Sn150 (SOMATOM Force). All patients received 60 mL of contrast medium (Lopamiro 370 mgI/mL, Bracco Sine pharmaceutical, Shanghai, China) followed by 40 mL of saline solution, at a flow-rate of 4.5 mL/s. The bolus-tracking technique was used to trigger the start of image acquisition, with ROI placement in the ascending aorta, the ROI threshold was 180 HU. The delay time between reaching the threshold and the start of the cardiac CTA acquisition was 3 s.

### Image reconstruction

All images were reconstructed with a constant level of iterative reconstruction (SAFIRE 2 on SOMATOM Flash and ADMIRE 2 on SOMATOM Force), with a slice thickness of 0.75 mm and intervals of 0.5 mm. The auto-best-phase technique was applied to get a serial of auto-phase images, and an advanced VMI+ algorithm was used to get a serial of DE blended rendering images. Whole-cycle Images were acquired at 9% R-R intervals across the entire cardiac cycle from 1% to 100%.

### Analysis of CT data

Cardiac CTA images were analyzed by members from the CVH (China Valve Hangzhou) CoreLab, using 3mensio 10.1 software (3mensio Medical Imaging BV, Bilthoven, Netherlands). Early events were defined as PASTE found within one year after TAVI, while late events were found later than four years after TAVI, and persistent events were defined as found in at least two consecutive annual follow-up cardiac CTA.

#### Leaflet evaluation: HALT

HALT was defined as visually identified increased leaflet thickness with a typical meniscal appearance in at least two different multi-planar reformation projections and present on at least two different reconstruction time intervals [20–23]. The extent of leaflet thickening was graded on long-axis views, carefully aligned with the leaflet center of the transcatheter heart valve regarding involvement along the curvilinear leaflet beginning at the base, a classic 4-tier grading scale was used for scoring [23]: 0: non-HALT; 1: 1–25%; 2: 26–50%; 3: 51–75%; 4: 76–100%.

#### Aortic sinus evaluation: SFD

SFD was defined as a hypoattenuated filling defect in the aortic sinus both in axis and long-axis views of the sinus, in at least two different multi-planar reconstructed images. According to the number of aortic sinuses involved, the SFD was scored on a 3-point scale: 0: non-SFD; 1: SFD with only one sinus involved; 2: SFD with two sinuses involved; 3: SFD with three sinuses involved.

#### Prosthesis evaluation: PFD

PFD is defined as a hypoattenuated filling defect in the metallic support structure of prosthesis both in short-axis and long-axis views of the prosthesis. It must be observed on at least two multi-planar reconstructed images to determine whether there is PFD.

### Patient outcomes

All the patients underwent standardized annual follow-ups by three trained cardiologists. The primary outcome was a major adverse cardiovascular composite outcome (MACCO), defined as the composite of stroke, cardiac re-hospitalization, and bioprosthetic valve dysfunction (BVD), BVD was defined as transvalvular mean pressure gradient (PGmean) ≥ 20 mmHg [8]. The secondary outcomes, which included bioprosthetic hemodynamics deterioration and cardiac dysfunction, were assessed with TTE by two senior echocardiologists, using Phillips IE33 and EPIQ 7 C system (Philips Electronics Ltd., Eindhoven, The Netherlands), following the American Society of Echocardiography standards for echocardiography core laboratories [24]. We qualitatively and quantitatively assessed bioprosthetic hemodynamics deterioration with long-term PGmean and ΔPGmean, and assessed cardiac dysfunction with long-term LVEF and ΔLVEF. All the trained cardiologists and senior echocardiologists were blinded to cardiac CTA imaging data.

### Statistical analysis

Analyses were performed using IBM SPSS Statistics 26.0 in the study cohort overall according to subclinical thrombotic events. The continuous variables were compared using the Mann–Whitney test. The frequencies were compared using the chi-square test when the frequency of each group was equal to or greater than 5. Fisher’s precision probability test was used when a set of frequencies was less than 5. To explore the relationship between PASTE, Mann–Whitney test was used to analyze the effects of PFD on the scores of thrombotic events in the leaflet and sinus. Fisher’s precision probability test was used to analyze the relationship between persistent PASTE in the aortic sinus and leaflet. Spearman correlation was used to explore the relationship between the scores of SFD and HALT. To explore the relationship between PASTE and long-term prognosis, we first used univariate analysis (Mann–Whitney test for continuous variables, Chi-square test for categorical variables) to explore the effect of PASTE on long-term prognosis. Then, we used Kaplan–Meier curves to assess the survival probability for the PASTE since TAVR, and the MACCO from PASTE. We then ran a survival analysis using Univariable and multivariate Cox proportional hazards regression to assess the relationship between risk factors and PASTE, and whether PASTE was related to the risk of the MACCO. A multivariate linear regression model was then used to analyze the effect of PASTE and its persistence on long-term prognosis by regressing out gender, age, BMI, D-dimer, and valve type. Finally, the relationship between early PASTE and long-term prognosis was analyzed by performing Spearman correlation in continuous variables, while chi-square test was used in categorical variables. In all analyses, *p* < 0.05 were considered statistically significant.

## Results

**Fig. 1:**
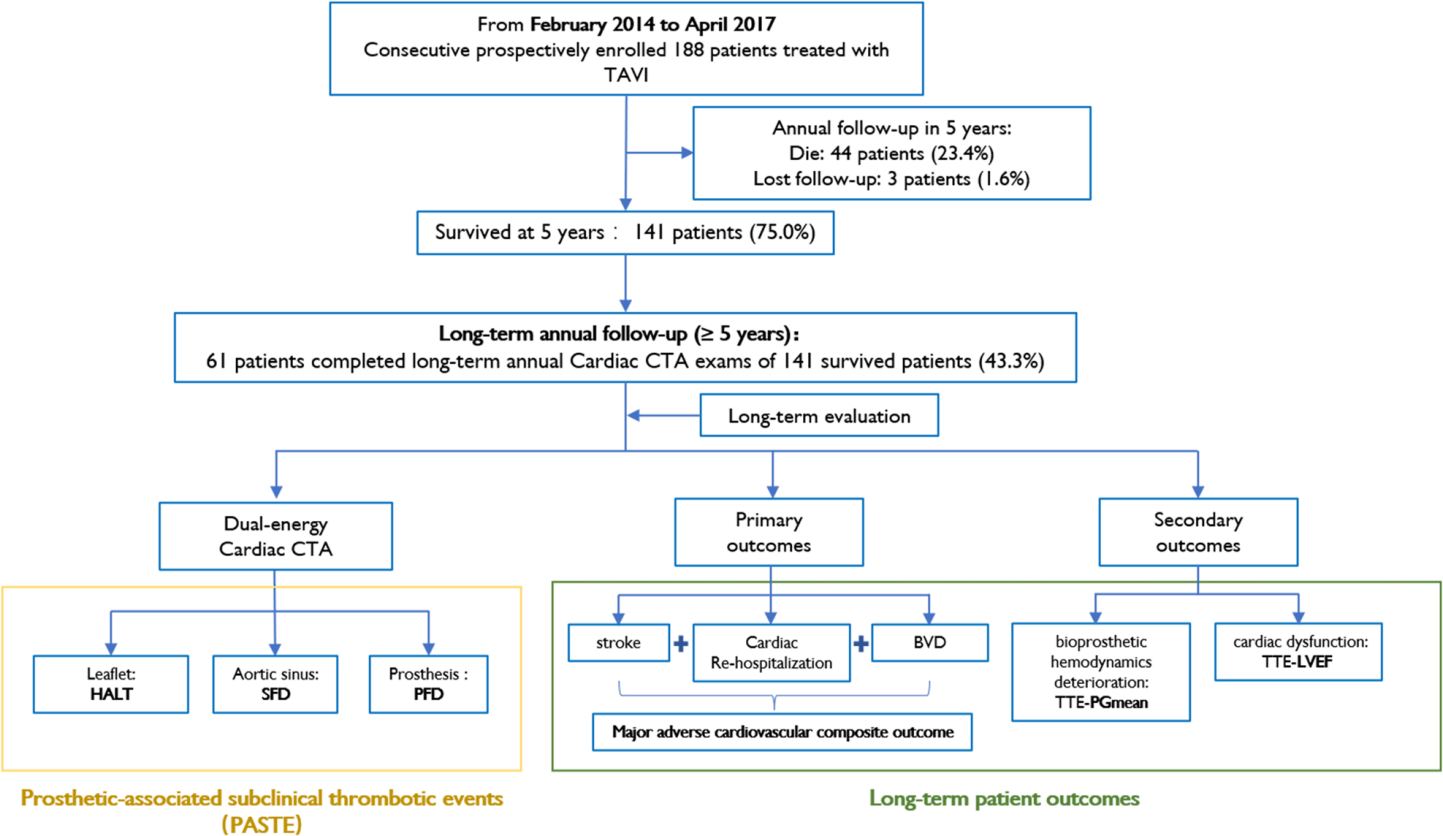
Study flow-chart. TAVI, transcatheter aortic valve implantation; PASTE, Prosthetic-associated subclinical thrombotic events; HALT, hypoattenuated leaflet thickening; SFD, sinus filling defect; PFD, prosthesis filling defect; BVD, bioprosthetic valve dysfunction; PGmean, mean transvalvular pressure gradient; LVEF, left ventricular ejection fraction

Between February 2014 and April 2017, 188 consecutive patients were treated with TAVI in our institution, among which 141 patients (75.0%) were confirmed to have survived for more than 5 years at annual follow-up, 44 patients (23.4%) died, and 3 patients (1.6%) were lost to follow-up. To evaluate long-term PASTE after TAVI, 61 (43.3%) patients who completed long-term annual cardiac CTA follow-up (≥ 5 years) were included in the study (Fig. 1). The other patients failed to complete annual cardiac CTA due to denial of repeat CTA, renal failure, telephone follow-up as an inability to come to the hospital due to some reason such as too far away from the hospital, inconvenient to walk, or epidemic control of COVID-19. The baseline and procedural characteristics of the study population are presented in Table 1.

**Table 1:** Clinical and procedural characteristics

Variable	Al l patients (*n* = 61)	non-HALT (*n* = 28)	HALT (*n* = 33)	P_1_	non-SFD (*n* = 38)	SFD (*n* = 23)	P_2_	non-PFD (*n* = 16)	PFD (*n* = 45)	P_3_
Age, y	74 [8]	73 [8]	75 [10]	0.566	73 [8]	76 [7]	0.095	73 [7]	75 [9]	0.786
Sex, *n* (%)				0.141			0.409			0.333
Female	28 (45.9)	10 (35.7)	18 (54.5)		19 (50.0)	9 (39.1)		9 (56.3)	19 (42.2)	
Male	33 (54.1)	18 (64.3)	15 (45.5)		19 (50.0)	14 (60.9)		7 (43.8)	26 (57.8)	
BMI, kg/m²	23.50 [4.50]	21.85 [3.65]	24.00 [4.00]	0.030*	23.45 [4.18]	23.50 [5.40]	0.829	23.75 [3.27]	23.30 [4.80]	0.577
STS score, %	4.42 (3.87)	4.44 (2.75)	4.3 (5.61)	0.439	4.44 (3.79)	4.30 (3.63)	0.623	3.07 (3.38)	4.43 (3.55)	0.115
NYHA Class	3 [1]	3 [1]	3 [1]	0.260	3 [1]	3 [1]	0.576	3 [1]	3 [1]	0.243
Hypertension, *n* (%)	36 (59)	14 (50)	22 (66.7)	0.187	22 (57.9)	14 (60.9)	0.819	10 (62.5)	26 (57.8)	0.741
Hypercholesterolemia, *n* (%)	20 (32.8)	8 (28.6)	12 (36.4)	0.518	12 (31.6)	8 (34.8)	0.796	5 (31.3)	15 (33.3)	0.879
Diabetes, *n* (%)	12 (19.7)	5 (17.9)	7 (21.2)	0.743	10 (26.3)	2 (8.7)	0.111	4 (25.0)	8 (17.8)	0.715
Atrial fibrillation, *n* (%)	8 (13.1)	4 (14.3)	4 (12.1)	1.000	5 (13.2)	3 (13.0)	1.000	3 (18.8)	5 (11.1)	0.422
Angina, *n* (%)	8 (13.1)	3 (10.7)	5 (15.2)	0.715	4 (10.5)	4 (17.4)	0.461	4 (25.0)	4 (8.9)	0.189
Previous PCI, *n* (%)	9 (14.8)	2 (7.1)	7 (21.2)	0.160	6 (15.8)	3 (13.0)	1.000	1 (6.3)	8 (17.8)	0.423
D-dimer, μg/L	430 [390]	425 [263]	480 [540]	0.144	475 [373]	390 [490]	0.448	355 [205]	480 [455]	0.027*
LVEF, %	57.1 (23.9)	56.9 (25.7)	59.6 (24.6)	0.208	58.8 (24.2)	55.1 (21.1)	0.958	62.9 (13.8)	54.3 (22.8)	0.053
Aortic valve area, cm²	0.58 [0.25]	0.58 [0.25]	0.58 [0.24]	0.931	0.59 [0.24]	0.57 [0.27]	0.743	0.59 [0.20]	0.57 [0.27]	0.749
Aortic valve velocity, m/s	4.8 [1.05]	4.97 [1.08]	4.77 [0.96]	0.405	4.80 [0.94]	4.85 [1.32]	0.783	5.17 [0.82]	4.77 [1.06]	0.051
Mean gradient, mmHg	54.0 [23.5]	60.0 [26.0]	52.0 [20.0]	0.250	53.5 [21.3]	57.0 [27.0]	0.688	64.5 [18.5]	52.0 [22.5]	0.079
Aortic valve type, *n* (%)				0.472			1.000			0.833
TAV	35 (57.4)	18 (64.3)	17 (51.5)		21 (55.3)	14 (60.9)		10 (62.5)	25 (55.6)	
BAV-type0	16 (26.2)	6 (21.4)	10 (30.3)		10 (26.3)	6 (26.1)		3 (18.75)	13 (28.9)	
BAV-type1	9 (14.8)	3 (10.7)	6 (18.2)		6 (15.8)	3 (13.0)		3 (18.75)	6 (13.3)	
Unknown	1 (1.6)	1 (3.6)	0 (0.0)		1 (2.6)	0 (0.0)		0 (0.0)	1 (2.2)	
Aortic valve calcification, mm³	1029.4 [1064.9]	1192.6 [1234.4]	832.5 [685.3]	0.179	873.5 [924.0]	1130.4 [1234.4]	0.698	1063.1 [832.8]	991.6 [1236.3]	0.763
Valve-in-valve, *n* (%)	5 (8.2)	4 (14.3)	1 (3.0)	0.170	3 (7.9)	2 (8.7)	1.000	1 (6.3)	4 (8.9)	1.000
Implanted Valve type, *n* (%)				0.031*			0.718			0.686
Self-expandable	52 (85.2)	27 (96.4)	25 (75.8)		33 (86.8)	19 (82.6)		13 (81.25)	39 (86.7)	
Mechanical-expandable	9 (14.8)	1 (3.6)	8 (24.2)		5 (13.2)	4 (17.4)		3 (18.75)	6 (13.3)	
Prosthesis size, *n* (%)				0.409			0.283			0.342
Small (21–24 mm)	13 (21.3)	6 (21.4)	7 (21.2)		6 (15.8)	7 (30.4)		2 (12.5)	11 (24.4)	
Medium (25–28 mm)	31 (50.8)	12 (42.9)	19 (57.6)		22 (57.9)	9 (39.1)		11 (68.8)	20 (44.4)	
Large (29–32 mm)	17 (27.9)	10 (35.7)	7 (21.2)		10 (26.3)	7 (30.4)		3 (18.8)	14 (31.1)	
Postdilatation, *n* (%)	23 (37.7)	13 (46.4)	10 (30.3)	0.195	13 (34.2)	10 (43.5)	0.469	4 (25.0)	19 (42.2)	0.250

Values are median with interquartile range unless otherwise specified. P1 showed the difference between non-HALT and HALT. P2 showed the difference between non-SFD and SFD. P3 showed the difference between non-PFD and PFD

Self-expandable Valve including CoreValue, Venus, Venus A, Microport; Mechanical-expandable Valve including Lotus; Unknown means Previous SAVR

*NYHA* New York Heart Association, *COPD* chronic obstructive pulmonary disease, *HALT* hypoattenuating leaflet thickening, *SFD* sinus filling defect, *PFD* prosthesis filling defect, *STS* Society of Thoracic Surgeons, *LVEF* left ventricular ejection fraction, *BMI* body mass index, *TAV* three-leafed aortic valve, *BAV* bicuspid aortic valve, *AVC* aortic valve calcification, *PCI* percutaneous coronary intervention, *CABG* coronary artery bypass grafting

*p < 0.05

### Prosthetic-associated subclinical thrombotic events (PASTE)

**Fig. 2:**
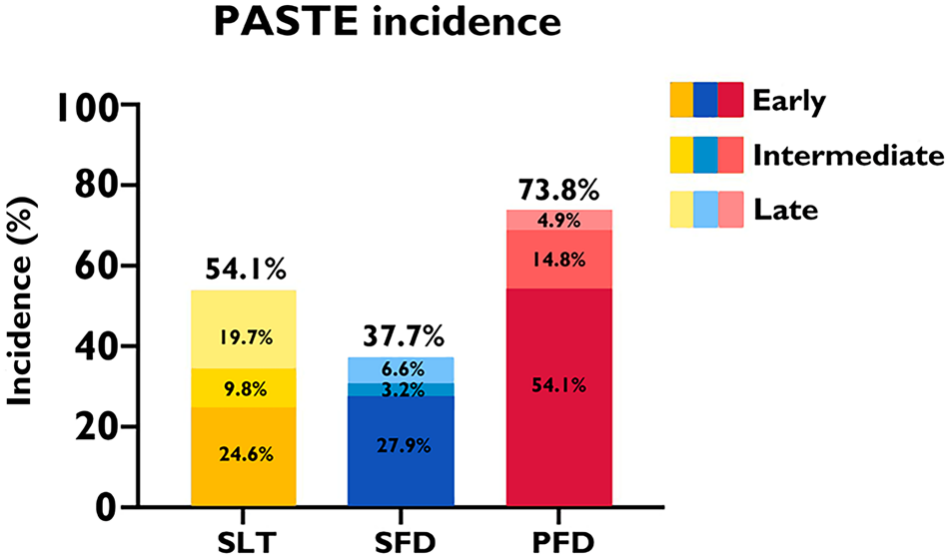
PASTE in cardiac CTA. Three types of PASTE are found in cardiac CTA after TAVI, including HALT (yellow bar), SFD (blue bar), and PFD (red bar). PASTE, Prosthetic-associated subclinical thrombotic events; HALT, hypoattenuated leaflet thickening; SFD, sinus filling defect; PFD, prosthesis filling defect

During the long-term follow-up by cardiac CTA, we found three types of PASTE, including HALT, SFD, and PFD (Fig. 2). The global view of PASTE is presented in Table 2. Over more than 5 years of long-term follow-up, the cumulative event incidence of HALT, SFD, and PFD were 54.1%, 37.7%, and 73.8% respectively, while early events (within one year) incidence was 24.6%, 27.9%, and 54.1% respectively, late events (later than four years) incidence was 19.7%, 6.6%, and 4.9% respectively, and persistent events (found in at least two consecutive annual follow-up) incidence was 18.0%, 29.5%, and 63.9%, respectively.

**Table 2:** Global view of PASTE

PASTE	Leaflet evaluation:	Aortic sinus evaluation:	Prosthesis evaluation:
	HALT	SFD	PFD
Non-events, *n* (%)	28 (45.9)	38 (62.3)	16 (26.2)
Events, *n* (%)	33 (54.1)	23 (37.7)	45 (73.8)
Events score	1 [3]	0 [1]	/
Early events, *n* (%)	15 (24.6)	17 (27.9)	33 (54.1)
Later events, *n* (%)	12 (19.7)	4 (6.6)	3 (4.9)
Occurrence time (y)	2 [4]	1 [2]	1 [1]
Persistent events, *n* (%)	11 (18.0)	18 (29.5)	39 (63.9)

Values are median with interquartile range unless otherwise specified. Persistent events were defined as PASTE that were found in at least two consecutive annual follow-up cardiac CTA, early events were found within one year after TAVI, and later events were found later than four years after TAVI

*PASTE* prosthetic-associated subclinical thrombotic events, *HALT* hypoattenuated leaflet thickening, *SFD* sinus filling defect, *PFD* prosthesis filling defect, *TAVI* transcatheter aortic valve implantation

#### Leaflet evaluation: HALT

**Fig. 3:**
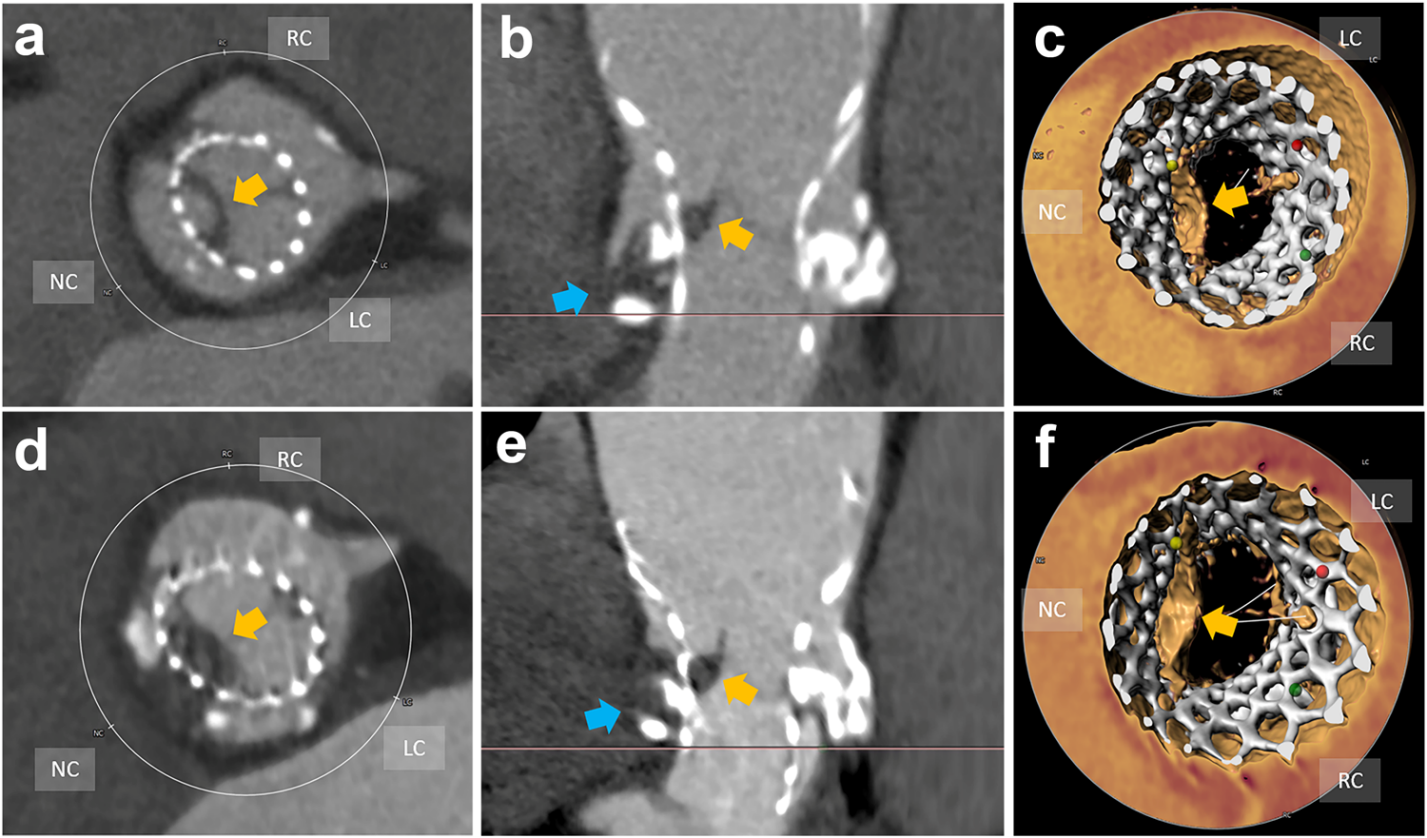
HALT score 2–3 and persistent SFD: **a** The non-coronary leaflet thickening (yellow arrow) on the short-axis view in 5-year follow-up cardiac CTA after TAVI, (**b**) The leaflet was hypoattenuated thickening confined to half of the leaflet (yellow arrow) on the long-axis view, and SFD was found in the non-coronary sinus (blue arrow), (**c**) thickening of the left leaflet (yellow arrow) on VRT reconstruction image. **d**, **e** The non-coronary leaflet thickening increased to about 3/4 of the leaflet (yellow arrow) in the 6-year CTA, SFD was also progressed (blue arrow). **f** Leaflet thickening is more obvious (yellow arrow) in the 6-year CTA than that in the 5-year CTA

On 50 Leaflets of 33 (54.1%) patients, HALT was found through the whole long-term follow-up, of which 11 (18.0%) of them (11/33, 33.3%) were persistent HALT, 15 (24.6%) were early HALT (15/33, 45.5%) and 12 (19.7%) were late HALT (12/33, 36.4%). The average scores of HALT are higher in early HALT (3.80 ± 1.90) than in late HALT (1.92 ± 1.26), while persistent HALT (3.91 ± 2.27) has the highest score. Some examples of HALT scores are shown in Fig. 3.

#### Aortic sinus evaluation: SFD

In 34 sinuses of 23 (37.7%) patients, SFD was found throughout the whole follow-up, of which non-coronary sinus involvement was found in 15 patients (65.2%). Left-coronary sinus involvement in 8 patients (34.8) and right-coronary sinus involvement in 11 patients (47.8%). 17 (27.9%) were early SFD (17/23, 73.9%) and 4 (6.6%) were late SFD (4/23, 17.4%), while 18 (29.5%) patients (18/23, 78.3%) were persistent SFD (Fig. 3).

#### Prosthesis evaluation: PFD

**Fig. 4:**
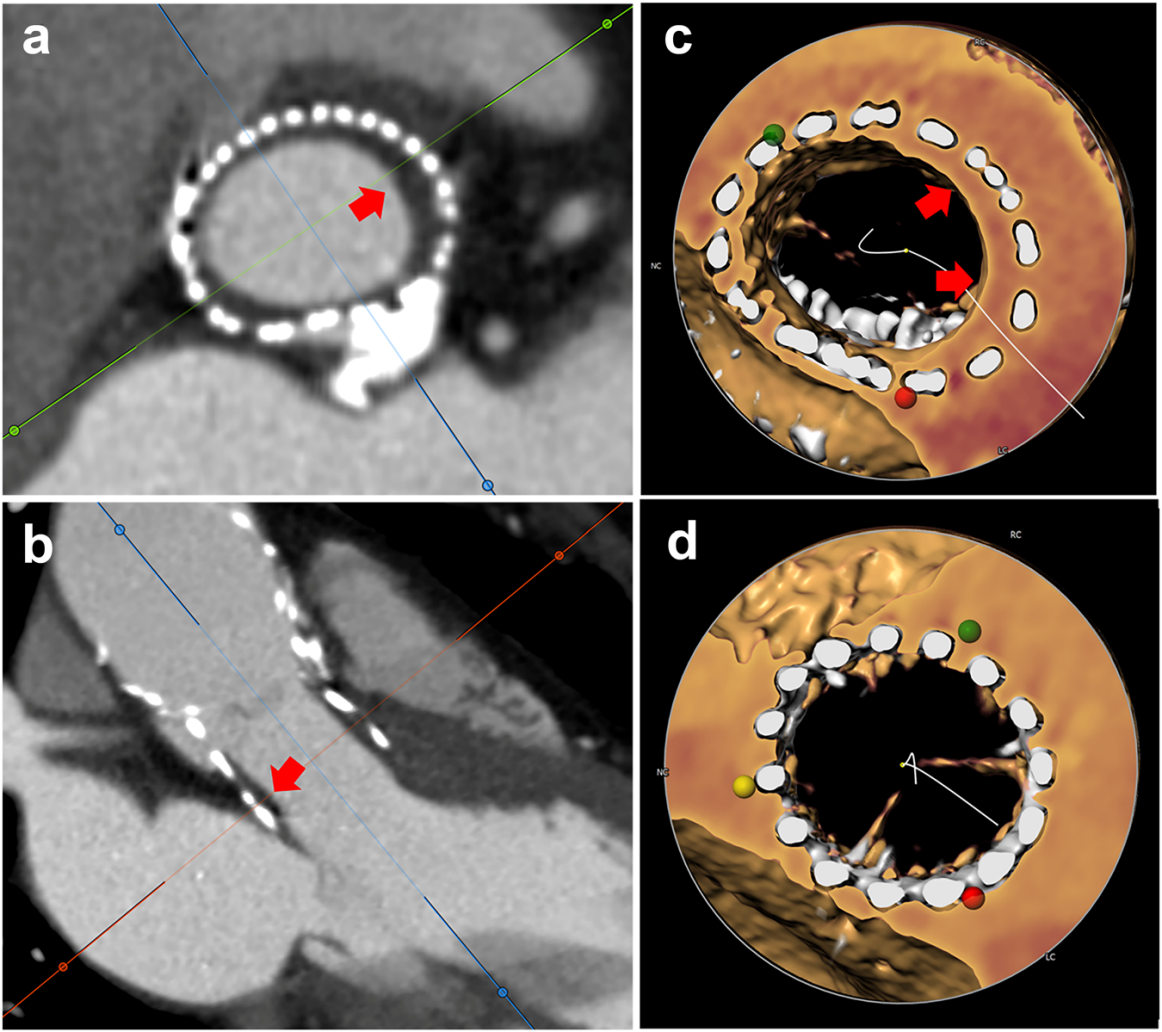
PFD: **a**, **b** Circumferential and asymmetric hypoattenuated filling defect (red arrow) within the prosthesis on the short- and long-axis view, (**c**) The luminal circumference of the prosthesis was encircled by soft tissue (red arrow). **d** No soft tissue coverage in the lumen of the prosthesis in another non-PFD patient

PFD (Fig. 4) was found in 45 (73.8%) patients through the whole cardiac CTA follow-up, 33 (54.1%) of them were early PFD (33/45, 73.3%), 3 (4.9%) were late PFD (3/45, 6.7%), while 39 (63.9%) were persistent PFD (39/45, 86.7%).

In the analysis of risk factors for PASTE, previous PCI (hazard ratio [HR]: 2.450; 95% CI: 1.046 to 5.740, *p* = 0.039), D-dimer (HR: 1.000; 95% CI: 1.000 to 1.000, *p* = 0.025), and implanted mechanical-expandable valve (HR: 2.417; 95% CI: 1.078 to 5.418, *p* = 0.032) might be risk factors for HALT in univariable analysis (See in Table S1). On adjusted multivariable analysis, BMI (HR: 1.124; 95% CI: 1.012 to 1.249, *p* = 0.030), Previous PCI (HR: 3.932; 95% CI: 1.563 to 9.894, *p* = 0.004), D-dimer (HR: 1.000; 95% CI: 1.000 to 1.000, *p* = 0.005), and implanted mechanical-expandable valve (HR: 3.126; 95% CI: 1.316 to 7.425, *p* = 0.010) might be risk factors for HALT (See in Table S2).

### Relationship between PASTE

SFD was significantly positively correlated with HALT (*r* = 0.34, *p* = 0.008), while PFD was not related to HALT (*p* = 0.550) and SFD (*p* = 0.435). In persistent PASTE, persistent HALT was significantly positively correlated with persistent SFD (*p* < 0.001).

### Patients outcomes

During the long-term follow-up at a median time of 63 months (IQR 60–73 months), MACCO occurred in 21 (34.4%) patients, of which 7 (11.5%) patients with stroke, 15 (24.6%) patients with cardiac re-hospitalization, and 4 (6.6%) patients with BVD, long-term primary and secondary outcomes were presented in Table 3.

**Table 3:** Long-term patient outcomes

	Al l (*n* = 61)	non-HALT (*n* = 28)	HALT (*n* = 33)	P_1_	non-SFD (*n* = 38)	SFD (*n* = 23)	P_2_	non-PFD (*n* = 16)	PFD (*n* = 45)	P_3_
Primary outcomes										
Major Adverse Cardiovascular Composite Outcome, *n* (%)	21 (34.4)	11 (39.3)	10 (30.3)	0.462	8 (21.1)	13 (56.5)	0.005*	5 (31.3)	16 (35.6)	0.756
Secondary outcomes										
Increased PGmean, *n* (%)	9 (14.8)	1 (3.6)	8 (24.2)	0.031*	4 (10.5)	5 (21.7)	0.278	4 (25.0)	5 (11.1)	0.224
PGmean, mmHg	9.0 [7.5]	8.0 [8.0]	9.0 [8.0]	0.606	9.0 [7.0]	8.0 [10.0]	0.994	7.5 [8.0]	9.0 [9.0]	0.521
ΔPGmean, mmHg	−4.0 [6.0]	−5.5 [6.0]	−3.0 [7.0]	0.064	−3.0 [6.0]	−4.0 [6.0]	0.429	−5.5 [9.0]	−3.0 [6.0]	0.505
Decreased LVEF, *n* (%)	33 (54.1)	13 (46.4)	20 (60.6)	0.268	19 (50.0)	14 (60.9)	0.409	11 (68.8)	22 (48.9)	0.171
LVEF, %	62.3 (7.3)	62.5 (8.2)	62.3 (7.9)	0.643	62.9 (8.4)	61.7 (7.6)	0.308	62.4 (11.7)	62.2 (6.3)	0.857
ΔLVEF, %	−0.4 (14.5)	1.7 (12.3)	−2.3 (17.4)	0.190	0.5 (10.7)	−1.1 (23.8)	0.970	−3.4 (12.1)	1.1 (14.3)	0.129

Values are median with interquartile range unless otherwise specified. P1 showed the difference between non-HALT and HALT. P2 showed the difference between non-SFD and SFD. P3 showed the difference between non-PFD and PFD

The major adverse cardiovascular composite outcome was a composite of stroke, cardiac re-hospitalization, and BVD

*PASTE* prosthetic-associated subclinical thrombotic events, *HALT* hypoattenuating leaflet thickening, *SFD* sinus filling defect, *PFD* prosthesis filling defect, *PGmean* mean transvalvular pressure gradient, *LVEF* left ventricular ejection fraction

**Fig. 5:**
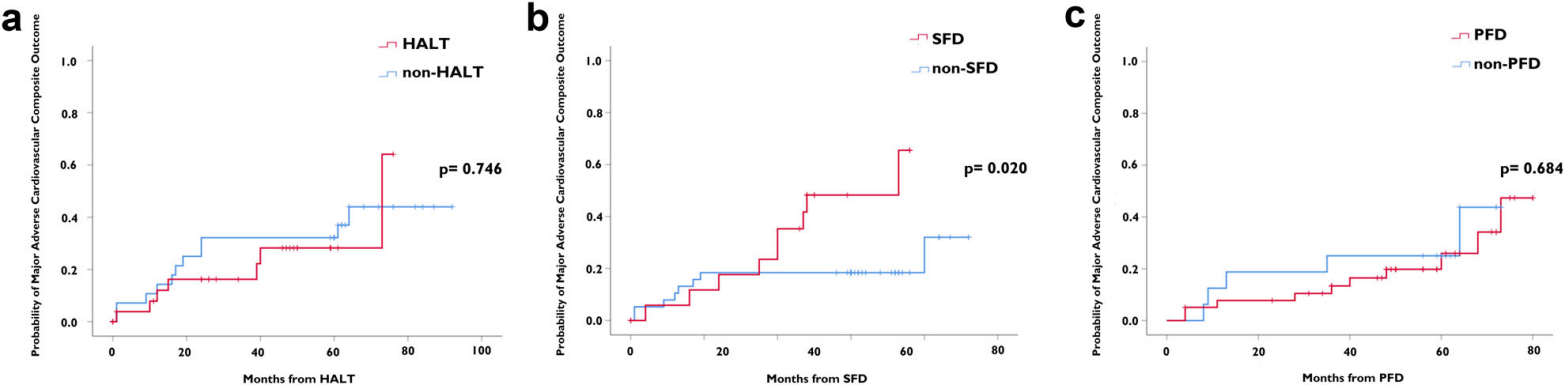
Kaplan-Meier analysis of MACCO According to PASTE, (**b**) SFD (*p* = 0.020) was associated with a higher incidence of the MACCO, while (**a**) HALT (*p* = 0.746) and (**c**) PFD (*p* = 0.684) did not have a statistically significant increase in MACCO incidence. The MACCO was a composite of stroke, cardiac re-hospitalization, and BVD. MACCO, major adverse cardiovascular composite outcome; PASTE, prosthetic-associated subclinical thrombotic events; HALT, hypoattenuating leaflet thickening; SFD, sinus filling defect; PFD, prosthesis filling defect

In the primary outcome, SFD and early SFD were significantly associated with the MACCO of stroke, cardiac re-hospitalization, and BVD (SFD: *p* = 0.005; early SFD: *p* = 0.018). Kaplan-Meier analysis also suggested that SFD (*p* = 0.020) was associated with a higher incidence of the MACCO (Fig. 5). Multivariate analysis of Cox proportional hazards regression suggested that SFD (HR: 2.870; 95% CI: 1.010 to 8.154, *p* = 0.048) was a predictor for the MACCO (Table 4).

**Table 4:** Multivariable predictors of major adverse cardiovascular composite outcome

Variable	Leaflet evaluation: HALT				Aortic sinus evaluation: SFD				Prosthesis evaluation: PFD			
	*n*	β	*P*	HR (95% CI)	*n*	β	*P*	HR (95% CI)	*n*	β	*P*	HR (95% CI)
PASTE												
No	28	−0.473	0.410	0.623 (0.202, 1.920)	38	1.054	0.048*	2.870 (1.010, 8.154)	16	−0.170	0.763	0.844 (0.281, 2.537)
Yes	30				19				39			
Sex												
Female	27	−0.140	0.785	0.870 (0.319, 2.371)	27	0.142	0.789	1.152 (0.408, 3.251)	26	0.246	0.668	1.279 (0.415, 3.937)
Male	31				30				29			
Age (y)	58	0.045	0.400	1.046 (0.942, 1.162)	57	0.032	0.521	1.302 (0.937, 1.136)	55	0.031	0.577	1.031 (0.926, 1.148)
BMI (kg/m²)	58	0.051	0.478	1.052 (0.914, 1.211)	57	0.007	0.925	1.007 (0.873, 1.162)	55	0.035	0.673	1.036 (0.879, 1.221)
D-dimer (μg/L)	58	< 0.001	0.819	1.000 (0.999, 1.000)	57	< 0.001	0.485	1.000 (0.999, 1.000)	55	< 0.001	0.614	1.000 (0.998, 1.001)
Valve type												
Self-expandable	50	0.856	0.245	2.353 (0.556, 9.953)	48	0.235	0.700	1.265 (0.384, 4.167)	47	0.020	0.976	1.021 (0.267, 3.902)
Mechanical-expandable	8				9				8			

The major adverse cardiovascular composite outcome was a composite of stroke, cardiac re-hospitalization, and BVD

*PASTE* prosthetic-associated subclinical thrombotic events, *HALT* hypoattenuating leaflet thickening, *SFD* sinus filling defect, *PFD* prosthesis filling defect

In the secondary outcomes, our study found HALT was significantly associated with increased PGmean (*p* = 0.031). In multivariate linear regression analysis adjusted for gender, age, and clinical characteristics that differed between groups (BMI, D-dimer, and planted valve type), long-term ΔPGmean was significantly correlated with persistent HALT (β = 0.38, *p* = 0.035), while long-term ΔLVEF was significantly negatively correlated with SFD (β = −0.39, *p* = 0.041) (Table 5). Furthermore, early SFD was significantly negatively correlated with long-term LVEF (*r* = −0.50, *p* = 0.041) and long-term ΔLVEF (*r* = −0.53, *p* = 0.030).

**Table 5:** The result of Multivariate linear regression analysis of Secondary outcomes

	ΔPGmean (mmHg)				ΔLVEF (%)			
	Un-standardized β	Standardized β	*p*	VIF	Un-standardized β	Standardized β	*p*	VIF
Age, y	−0.028 (−0.283, 0.227)	−0.03	0.828	1.37	0.055 (−0.597, 0.707)	0.02	0.866	1.37
Sex	−2.440 (−4.746, −0.135)	−0.25	0.038*	1.12	3.853 (−2.043, 9.748)	0.17	0.195	1.12
BMI, kg/m²	0.265 (−0.079, 0.610)	0.18	0.128	1.12	−0.402 (−1.282, 0.479)	−0.12	0.364	1.12
D-dimer, μg/L	−0.000 (−0.001, 0.001)	−0.01	0.957	1.33	0.002 (−0.000, 0.004)	0.23	0.102	1.33
Valve type	5.338 (1.681, 8.996)	0.38	0.005*	1.42	−9.058 (−18.412, 0.296)	−0.28	0.057	1.42
HALT	−0.487 (−1.257, 0.283)	−0.19	0.210	1.87	0.534 (−1.435, 2.503)	0.09	0.588	1.87
SFD	−1.052 (−3.146, 1.041)	−0.17	0.317	2.38	−5.603 (−10.957, −0.250)	−0.39	0.041*	2.38
PFD	−2.700 (−6.845, 1.445)	−0.24	0.197	2.81	7.624 (−2.976, 18.225)	0.29	0.155	2.81
Persistent HALT	4.828 (0.349, 9.308)	0.38	0.035*	2.51	2.168 (−9.287, 13.624)	0.07	0.705	2.51
Persistent SFD	0.157 (−3.717, 4.032)	0.02	0.935	2.64	7.510 (−2.398, 17.417)	0.30	0.134	2.64
Persistent PFD	2.863 (−0.996, 6.722)	0.28	0.142	2.90	−7.201 (−17.069, 2.667)	−0.30	0.149	2.90

For Long-Term ΔPGmean (mmHg), Durbin-Watson = 1.91, Adjusted R² = 0.28, Constant = −12.16. For Long-Term ΔLVEF (%), Durbin-Watson = 1.66, Adjusted R² = 0.12, Constant = 13.64. β means standardized β value

*PGmean* mean transvalvular pressure gradient, *LVEF* left ventricular ejection fraction, *BMI* body mass index, *HALT* hypoattenuated leaflet thickening, *SFD* sinus filling defect, *PFD* prosthesis filling defect, *VIF* variance inflation factor

*p < 0.05

## Discussion

The main findings of our study are as follows: (1) There were three types of PASTE found in cardiac CTA after TAVI, including HALT, SFD, and PFD, cumulative event rates of them over 5 years long-term follow-up were 54.1%, 37.7%, and 73.8%, respectively. Among them, SFD showed a significant positive correlation with HALT (*p* = 0.008). (2) In the primary outcome, SFD and early SFD were significantly associated with the MACCO of stroke, cardiac re-hospitalization, and BVD (SFD: *p* = 0.005; early SFD: *p* = 0.018), and SFD was a major predictor of the MACCO (HR: 2.870; 95% CI: 1.010 to 8.154, *p* = 0.048). (3) In the secondary outcomes, HALT was significantly associated with increased PGmean (*p* = 0.031), while persistent HALT was significantly correlated with long-term ΔPGmean (β = 0.38, *p* = 0.035), which implied that HALT was associated with long-term hemodynamic deterioration in patients after TAVI. On the other hand, SFD was significantly negatively correlated with ΔLVEF (β = −0.39, *p* = 0.041), which implied that SFD was associated with long-term cardiac dysfunction. Moreover, early SFD was significantly negatively correlated with LVEF and ΔLVEF (LVEF: *r* = −0.50, *p* = 0.041; ΔLVEF: *r* = −0.53, *p* = 0.030).

Over the last few years, it has been recognized that transcatheter heart valve thrombosis is often under-diagnosed and may be a potential cause of valvular dysfunction [25], while HALT was considered to be the first stage of structural valve degeneration, as it may progress in severity to clinical valve thrombosis and cause significant leaflet dysfunction, which typically leads to recurrent symptoms of aortic stenosis [26]. In the PARTNER 3 and Evolut randomized trials, the incidence of HALT in cardiac CTA after TAVI or SAVR varied from 10–16% at 30 days and increased to 24–30% at 1 year [8, 23, 27, 28], which is described as hypoattenuated leaflet thickening (HALT) in cardiac CTA. In our study, the incidence of early HALT (within 1 year after TAVI) was 24.6%, which was consistent with the previous study [8, 9]. In the long-term follow-up of our study, the incidence of HALT was 54.1%, which means that there are still many HALT events occurring one year after TAVI. However, whether these HALT, which occur one year after TAVI would have some impacts on the long-term clinical outcome of these patients needs to be verified with more multicenter and prospective data. A 3-year follow-up study showed that HALT was associated with symptomatic hemodynamic valve deterioration [26]. Our study found that HALT was significantly associated with increased PGmean after TAVI (*p* = 0.031), and persistent HALT was significantly associated with long-term ΔPGmean (β = 0.38, *p* = 0.035), which also demonstrated that HALT was associated with long-term hemodynamic deterioration in patients after TAVI. Moreover, in the analysis of risk factor predictor of PASTE, we found that BMI (HR: 1.124; 95% CI: 1.012 to 1.249, *p* = 0.030), Previous PCI (HR: 3.932; 95% CI: 1.563 to 9.894, *p* = 0.004), D-dimer (HR: 1.000; 95% CI: 1.000 to 1.000, *p* = 0.005), and implanted mechanical-expandable valve (HR: 3.126; 95% CI: 1.316 to 7.425, *p* = 0.010) might be the high-risk factors for HALT. In the future, large-sample cohort studies can be conducted based on these high-risk factors to further evaluate their impact on HALT, and their possible mechanisms, to reduce the incidence of HALT and improve the prognosis of patients after TAVI.

In our study, we found SFD to be another PASTE that deserves great attention. It was associated with the long-term MACCO of stroke, cardiac re-hospitalization, and BVD (*p* = 0.005), and was the most important predictor of the long-term MACCO (HR: 2.870; 95% CI: 1.010 to 8.154, *p* = 0.048). SFD was also associated with long-term cardiac dysfunction of reduced LVEF (β = −0.39, *p* = 0.041) in patients after TAVI. In addition, that SFD was associated with HALT (*p* = 0.008). Therefore, SFD may be a potential target for initiating anticoagulant treatment after TAVI. Similar to previous findings, this study showed that SFD was mostly located at the bottom of the sinuses and extended upward toward the sinotubular junction [16]. As we know, TAVI is implanted with a prosthesis, which is a net-like metal stent structure above the leaflets to ensure coronary artery blood perfusion, and a membrane-covered structure below the leaflets to reduce the possibility of paravalvular leakage. Perhaps we can assume that the mechanism of SFD related to HALT may be due to the special structure of TAVI implanted prosthesis—when thrombi in the sinus overpass the native leaflet outside the prosthetic from bottom to top, it may pass through the net-like structure of the prosthesis and then involve the implanted leaflet, which may need to be further confirmed by animal experiments, autopsy, or surgery. Identical to other studies [16, 29], SFD was most commonly involved in the non-coronary sinus (15/23, 65.2%) in our study. This may be because blood stays longer in the noncoronary sinus than in the other two sinuses. Moreover, blood flow at the base of the sinus of Valsalva was stagnant throughout the cardiac cycle [16, 29]. Therefore, altered hemodynamics in the sinuses may be a significant cause of SFD. In the future, studies on the mechanism of the formation of SFD are needed. Furthermore, randomized controlled trials (RCT) studies based on SFD as a potential target for anticoagulation therapy are needed, which may provide important evidence to improve guidelines on the management of antithrombotic therapy after TAVI.

Our study showed that PFD was observed in 73.8% of patients through long-term follow-up, which was obviously higher than that of the surgical mechanical valve (19%) [18]. This may be because the structure of the transcatheter valve is longer than surgical mechanical valve, so the sub-valvular structure of the prosthesis has more contact area with the left ventricular outflow tract and the left ventricular wall, some of which may even be inserted into the left-ventricular wall, which makes it more likely to develop thrombosis or pannus in the prosthesis. It might be noted that we observed PFD after TAVI as an imaging manifestation, without identifying thrombus or pannus, which can be further studied in future research.

**Fig. 6:**
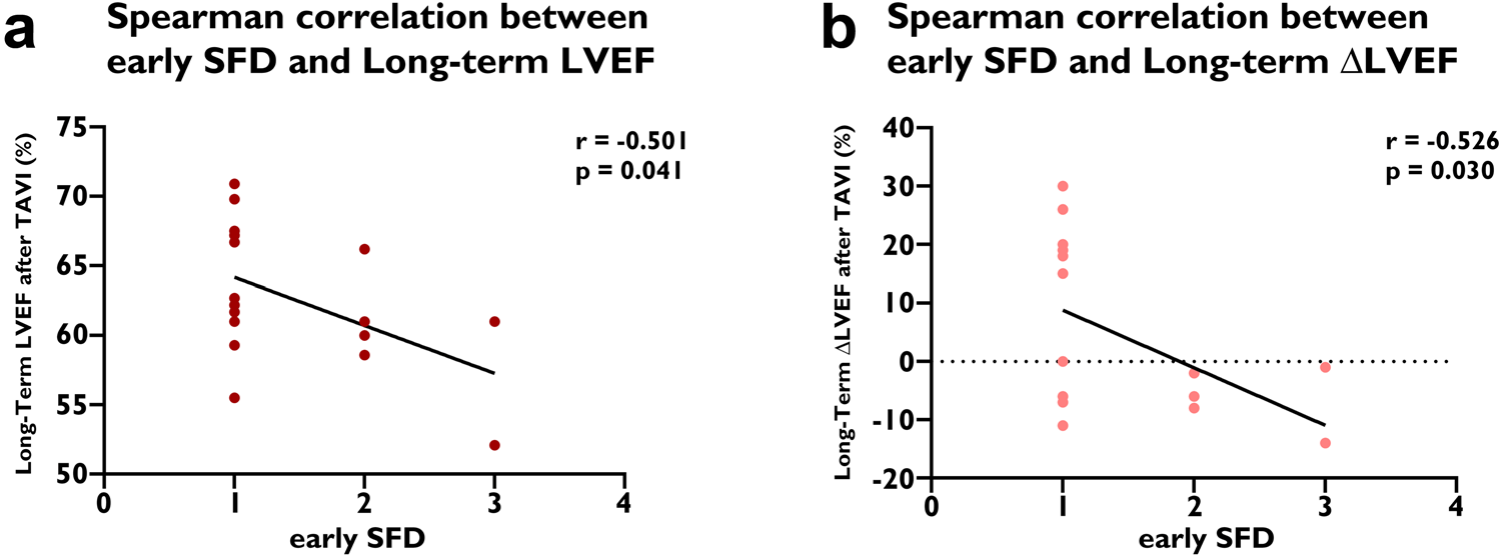
Early SFD and Long-Term LVEF/ΔLVEF. Early SFD was associated with (**a**) Long-Term LVEF (*p* = 0.041) and (**b**) Long-Term ΔLVEF (*p* = 0.030). TAVI, transcatheter aortic valve implantation; SFD, sinus filling defect; LVEF, left ventricular ejection fraction

Furthermore, this study also focused on early PASTE. Previous studies on early HALT (30 days to 1 year) have shown that HALT was associated with mortality, TIA and stroke, structural valve degeneration, or symptomatic hemodynamic valve deterioration [9, 11–14]. Our data showed that early SFD was also associated with long-term patient outcomes, as early SFD was associated with the MACCO (stroke, cardiac re-hospitalization, and BVD: *p* = 0.018) and cardiac dysfunction (LVEF: *r* = −0.50, *p* = 0.041; ΔLVEF: *r* = −0.53, *p* = 0.030) (Fig. 6). It reminds us that we may need to pay attention to the impact of early PASTE, especially early SFD, and their impact on the long-term patient outcomes.

There is evidence that oral anticoagulation (OAC) or non-vitamin-K antagonist oral anticoagulant (NOAC) can effectively prevent and reverse leaflet thrombosis [8, 15, 20, 23, 30, 31], but may result in a higher risk of bleeding [15]. However, the current guidelines for the management of antithrombotic therapy in patients with valvular heart disease or undergoing TAVI do not recommend adjusting the antithrombotic strategy based on CT imaging findings of subclinical thrombotic events, until there is an associated hemodynamic deterioration [2, 32, 33]. Therefore, the subjects in our study did not adjust the antithrombotic strategy based on PASTE. The antithrombotic strategy for the patients in our cohort was based on standard antithrombotic management strategies for patients with heart valve disease. Details can be found in Table S3. However, as shown in this study, PASTE was associated with long-term adverse outcomes of the patients, especially the association between SFD and the MACCO in patients, suggesting that it may be a potential target for anticoagulation in patients after TAVI, which will require further studies.

In this study, although the patients were prospectively collected, the analysis was performed retrospectively, based on the long-term CTA findings and patient outcomes. As this study was an exploratory and observational study based on CTA findings, in order to preserve the integrity of the long-term CTA data to the extent possible, which caused a relatively large dropout rate. The final sample size of this study was relatively small and brought about a certain selection bias. Therefore, the generalizability of this study needed to be further validated with larger sample size studies and multi-center data. In addition, this study did not confirm by pathology whether the filling defect shown by cardiac CTA was a thrombus. However, our study showed that these cardiac CTA findings of PASTE do have some impact on the long-term outcomes of patients, and have clinical significance. Therefore, it is hoped that PASTE after TAVI will be given more attention, so that more studies can be developed around PASTE, including more basic studies in fields such as pathology, biology, and fluid mechanics.

## Conclusions

During the long-term follow-up of more than 5 years, PASTE including HALT, SFD, and PFD were found in cardiac CTA after TAVI. PASTE were associated with long-term major adverse outcomes, bioprosthetic hemodynamics deterioration, and cardiac dysfunction. In particular, SFD was a major predictor of long-term major adverse outcomes in patients after TAVI, and therefore it may be a potential anticoagulation target in patients after TAVI.

### Supplementary information

Electronic Supplementary Material

## Data Availability

The data and material underlying this article will be shared on reasonable request to the corresponding author.

## Abbreviations

BVD: Bioprosthetic valve dysfunction
CTA: Computed tomography angiography
HALT: Hypoattenuated leaflet thickening
LVEF: Left ventricular ejection fraction
MACCO: Major adverse cardiovascular composite outcome
PASTE: Prosthetic-associated subclinical thrombotic events
PFD: Prosthesis filling defect
PGmean: Mean pressure gradient
SFD: Sinus filling defect
TAVI: Transcatheter aortic valve implantation
