# Supplementary material for: Long-term prosthetic-associated subclinical thrombotic events evaluation by cardiac CTA after transcatheter aortic valve implantation: incidence and outcomes
Source: Insights Imaging. 2024 May 30;15:125. doi: 10.1186/s13244-024-01681-0 (PMC11139807; doi:10.1186/s13244-024-01681-0)
Supplement: Supplementary file 1 — Electronic Supplementary Material [file 13244_2024_1681_MOESM1_ESM.pdf]

Long-term prosthetic-associated subclinical thrombotic events evaluation by cardiac CTA after transcatheter aortic valve implantation: incidence and outcomes

ELECTRONIC SUPPLEMENTARY MATERIAL

Table S1. Univariable Analysis of High-risk Factors of PASTE

Variable	Leaflet evaluation: HALT			Aortic sinus evaluation: SFD			Prosthesis evaluation: PFD		
	β	*p*	HR (95% CI)	β	*p*	HR (95% CI)	β	*p*	HR (95% CI)
Age	0.027	0.472	1.028 (0.954, 1.106)	0.086	0.064	1.090 (0.995, 1.194)	0.005	0.868	1.005 (0.944, 1.070)
Sex	-0.452	0.200	0.637 (0.319, 1.270)	0.340	0.427	1.405 (0.607, 3.253)	0.332	0.273	1.394 (0.769, 2.526)
BMI	0.094	0.052	1.099 (0.999, 1.209)	-0.010	0.868	0.990 (0.882, 1.112)	-0.013	0.779	0.987 (0.902, 1.080)
STS score	0.010	0.790	1.010 (0.937, 1.089)	0.006	0.899	1.006 (0.916, 1.105)	0.000	0.997	1.000 (0.940, 1.064)
NYHA Class	-0.176	0.479	0.838 (0.515, 1.365)	-0.100	0.741	0.905 (0.499, 1.640)	0.159	0.497	1.172 (0.741, 1.855)
Hypertension	0.632	0.090	1.881 (0.906, 3.905)	0.150	0.727	1.162 (0.500, 2.699)	-0.079	0.797	0.924 (0.508, 1.681)
Hypercholesterolemia	0.074	0.838	1.077 (0.529, 2.193)	0.049	0.911	1.050 (0.445, 2.479)	-0.017	0.956	0.983 (0.528, 1.831)
Diabetes	0.122	0.776	1.129 (0.489, 2.609)	-0.933	0.208	0.393 (0.092, 1.682)	-0.139	0.723	0.870 (0.403, 1.877)
Atrial fibrillation	0.067	0.900	1.070 (0.375, 3.051)	0.204	0.743	1.227 (0.362, 4.151)	-0.299	0.530	0.741 (0.291, 1.887)
Angina	0.094	0.847	1.098 (0.423, 2.852)	0.282	0.609	1.326 (0.451, 3.902)	-0.596	0.258	0.551 (0.197, 1.546)
Previous PCI	0.896	**0.039***	2.450 (1.046, 5.740)	-0.182	0.769	0.834 (0.247, 2.815)	0.363	0.360	1.437 (0.661, 3.125)
D-dimer	0.000	**0.025***	1.000 (1.000, 1.000)	0.000	0.146	1.000 (1.000, 1.000)	0.000	0.835	1.000 (1.000, 1.000)
LVEF	0.015	0.275	1.015 (0.988, 1.043)	0.004	0.777	1.004 (0.974, 1.036)	-0.007	0.485	0.993 (0.972, 1.013)
Aortic valve area	-0.143	0.877	0.867 (0.142, 5.282)	0.455	0.674	1.577 (0.189,13.174)	0.224	0.770	1.251 (0.279, 5.618)
Aortic valve velocity	-0.029	0.890	0.971 (0.643, 1.468)	-0.058	0.839	0.944 (0.538, 1.654)	-0.409	0.064	0.664 (0.431, 1.024)
Mean gradient	-0.010	0.407	0.990 (0.966, 1.014)	0.006	0.692	1.006 (0.978, 1.034)	-0.009	0.373	0.991 (0.971,1.011)
Aortic valve type	0.249	0.234	1.283 (0.851, 1.935)	-0.049	0.860	0.953 (0.555, 1.636)	-0.009	0.962	0.991 (0.672, 1.461)
Aortic valve calcification	0.000	0.142	1.000 (0.999, 1.000)	0.000	0.440	1.000 (1.000, 1.001)	0.000	0.702	1.000 (1.000, 1.001)
Valve-in-valve	-1.315	0.196	0.268 (0.037, 1.968)	0.140	0.850	1.151 (0.269, 4.927)	0.125	0.811	1.134 (0.405, 3.174)
Implanted Valve type	0.882	**0.032***	2.417 (1.078, 5.418)	0.414	0.455	1.513 (0.511, 4.477)	0.022	0.961	1.022 (0.428, 2.438)
Prosthesis size	-0.298	0.223	0.743 (0.460, 1.198)	-0.386	0.214	0.680 (0.370, 1.249)	-0.062	0.776	0.940 (0.612, 1.444)
Post-dilatation	-0.471	0.215	0.624 (0.296, 1.315)	0.428	0.313	1.535 (0.668, 3.525)	0.348	0.253	1.416 (0.780, 2.573)

Table S2. Multivariable Analysis of High-risk Factors of PASTE

Variable	Leaflet evaluation: HALT			Aortic sinus evaluation: SFD			Prosthesis evaluation: PFD		
	β	*p*	HR (95% CI)	β	*p*	HR (95% CI)	β	*p*	HR (95% CI)
Age	0.014	0.922	1.015 (0.937, 1.099)	0.081	0.087	1.085 (0.988, 1.190)	-0.001	0.971	0.999(0.937, 1.065)
Sex	-0.214	0.582	0.807 (0.376, 1.731)	0.335	0.464	1.398 (0.570, 3.427)	0.342	0.280	1.407 (0.757, 2.615)
BMI	0.117	**0.030***	1.124 (1.012, 1.249)	-0.001	0.982	0.999 (0.880, 1.133)	0.000	0.996	1.000 (0.906, 1.105)
Previous PCI	1.369	**0.004***	3.932 (1.563, 9.894)	0.044	0.946	1.045 (0.292, 3.738)	0.361	0.377	1.435 (0.645, 3.193)
D-dimer	0.000	**0.005***	1.000 (1.000, 1.000)	0.000	0.299	1.000 (1.000, 1.000)	0.000	0.655	1.000 (1.000, 1.000)
Implanted Valve type	1.140	**0.010***	3.126 (1.316, 7.425)	0.571	0.322	1.769 (0.292, 3.738)	0.065	0.886	1.067 (0.438, 2.601)

Table S3. PASTE and antithrombotic therapy

	Al l (n=61)	non-HALT (n=28)	HALT (n=33)	P_1	non-SFD (n=38)	SFD (n=23)	P_2	non-PFD (n=16)	PFD (n=45)	P_3
**baseline**										
Antiplatelet, n (%)	61 (100.0)	28 (100.0)	33 (100.0)	_	38 (100.0)	23 (100.0)	_	16 (100.0)	45 (100.0)	_
Anticoagulation, n (%)	8 (13.1)	4 (14.3)	4 (12.1)	1.000	4 (10.5)	4 (17.4)	0.461	3 (18.8)	5 (11.1)	0.422
**1 month after TAVI**										
Antiplatelet, n (%)	61 (100.0)	28 (100.0)	33 (100.0)	_	38 (100.0)	23 (100.0)	_	16 (100.0)	45 (100.0)	_
Anticoagulation, n (%)	9 (14.8)	4 (14.3)	5 (15.2)	1.000	4 (10.5)	5 (21.7)	0.278	2 (12.5)	7 (15.6)	1.000
**1 year after TAVI**										
Antiplatelet, n (%)	53 (86.9)	26 (92.9)	27 (81.8)	0.269	36 (94.7)	17 (73.9)	**0.044**	14 (87.5)	39 (86.7)	1.000
Anticoagulation, n (%)	9 (14.8)	3 (10.7)	6 (18.2)	0.488	4 (10.5)	5 (21.7)	0.278	2 (12.5)	7 (15.6)	1.000
**2 years after TAVI**										
Antiplatelet, n (%)	46 (75.4)	19 (67.6)	27 (81.8)	0.207	29 (76.3)	17 (73.9)	0.833	12 (75.0)	34 (75.6)	1.000
Anticoagulation, n (%)	11 (18.0)	6 (21.4)	5 (15.2)	0.525	6 (15.8)	5 (21.7)	0.558	3 (18.8)	8 (17.8)	1.000
**3 years after TAVI**										
Antiplatelet, n (%)	46 (75.4)	20 (71.4)	26 (78.8)	0.506	29 (76.3)	17 (73.9)	0.833	12 (75.0)	34 (75.6)	1.000
Anticoagulation, n (%)	10 (16.4)	5 (17.9)	5 (15.2)	0.776	5 (13.2)	5 (21.7)	0.380	2 (12.5)	8 (17.8)	1.000
**4 years after TAVI**										
Antiplatelet, n (%)	43 (70.5)	19 (67.9)	24 (72.7)	0.678	28 (73.7)	15 (65.2)	0.482	12 (75.0)	31 (68.9)	0.757
Anticoagulation, n (%)	13 (21.3)	6 (21.4)	7 (21.2)	0.984	4 (10.5)	9 (39.1)	**0.012**	1 (6.3)	12 (26.7)	0.153
**5 years after TAVI**										
Antiplatelet, n (%)	38 (62.3)	18 (64.3)	20 (60.6)	0.768	26 (68.4)	12 (52.2)	0.204	11 (68.8)	27 (60.0)	0.535
Anticoagulation, n (%)	12 (19.7)	4 (14.3)	8 (24.2)	0.519	4 (10.5)	8 (34.8)	**0.043**	1 (6.3)	11 (24.4)	0.156

PASTE: prosthetic-associated subclinical thrombotic events; HALT: hypoattenuating leaflet thickening; SFD: sinus filling defect; PFD: prosthesis filling defect; TAVI: transcatheter aortic valve implantation.
